# Associations of Vascular Risk Factors, *APOE* and *TOMM40* Polymorphisms With Cognitive Function in Dementia-Free Chinese Older Adults: A Community-Based Study

**DOI:** 10.3389/fpsyt.2021.617773

**Published:** 2021-03-15

**Authors:** Wenjun Gui, Chengxuan Qiu, Qi Shao, Juan Li

**Affiliations:** ^1^CAS Key Laboratory of Mental Health, Center on Aging Psychology, Institute of Psychology, Chinese Academy of Sciences, Beijing, China; ^2^Department of Psychology, University of Chinese Academy of Sciences, Beijing, China; ^3^Department of Neurology, Shandong Provincial Hospital Affiliated to Shandong University, Jinan, China; ^4^Aging Research Center and Center for Alzheimer Research, Department of Neurobiology, Care Sciences and Society, Karolinska Institutet and Stockholm University, Stockholm, Sweden

**Keywords:** aging, vascular risk factors, *APOE*, *TOMM40*, cognitive function, population-based study

## Abstract

**Objective:** The associations of vascular risk factors (VRFs), apolipoprotein E (*APOE*), and translocase of outer mitochondrial membrane 40 (*TOMM40*) with cognitive function have been investigated mostly in western societies. In the present study, we sought to examine the associations of VRFs [i.e., current smoking, current drinking, physical inactivity, obesity, total cholesterol (TC), triglycerides (TG), low-density lipoprotein cholesterol (LDL-C), high-density lipoprotein cholesterol (HDL-C), diabetes, and hypertension] and variants located in *APOE* (ε2/3/4) and *TOMM40* (rs2075650) with global cognitive function in Chinese older adults, with a focus on their potential interactions.

**Methods:** This is a cross-sectional study that included 422 permanent residents (mean age 69.2 years, 54.3% female) living in Beijing, who were free of dementia. Data were collected through interviews, clinical examinations, and laboratory tests. The two genetic polymorphisms were genotyped, and participants were dichotomized as carriers vs. non-carriers of *APOE* ε4 or *TOMM40* G. Global cognitive function was assessed with the Mini-Mental State Examination (MMSE). Data were analyzed with multivariable linear regression models.

**Results:** Physical inactivity and diabetes were independently associated with a lower MMSE score (all *p* < 0.05). When four putative VRFs (i.e., current smoking, physical inactivity, high LDL-C, and diabetes) were aggregated, an increasing number of having these factors was associated with a decreasing MMSE score in a dose–response manner (*p* = 0.001). *TOMM40* polymorphisms, independent of the *APOE* ε4 allele, interacted with aggregated VRFs to influence cognitive performance, such that having one or more of these VRFs was particularly detrimental to the cognition of *TOMM40* carriers. Further analyses revealed interactions of the *TOMM40* polymorphism with (i) physical inactivity and (ii) diabetes, such that having either physical inactivity or diabetes in combination with carrying a *TOMM40* G allele, compared to having neither, was significantly associated with a markedly lower MMSE score (all *p* < 0.05).

**Conclusion:** This study provides some evidence supporting the association of vascular risk factors with poor cognitive performance among dementia-free Chinese older adults and further revealed their interactions with the *TOMM40* polymorphism. The results underscore the vulnerability of global cognitive function to VRFs, which could be reinforced by carrying the *TOMM40* rs2075650 G allele. These findings have potential implications for developing tailored intervention programs to maintain cognitive function.

## Introduction

The proportion of older people has been growing rapidly in China over the past half-century, alongside social and economic development. In 2010, there were 178 million people aged 60 years and older in China, accounting for ~13.3% of the total population. Therefore, age-related disorders such as Alzheimer's disease (AD) and dementia have posed a tremendous burden to individuals and the society at large in China (1, 2). Recently, great efforts have been made to identify modifiable risk factors for early-stage cognitive disorders, such as mild cognitive impairment, which may help identify high-risk individuals and further facilitate the development of interventions to delay the onset of dementia. The associations of individual vascular risk factors (VRFs) and their aggregation with poor cognitive function have been well-established in western countries (3, 4). In China, some studies have reported the associations of individual VRFs (e.g., hypertension and diabetes) with cognitive impairment (5, 6). However, very few studies have examined the association of aggregated VRFs with global cognitive impairment. Modifiable or manageable VRFs often coexist among the elderly, and the concurrent presence of multiple VRFs plays a critical role in age-related cognitive decline (7). Thus, exploring the association of aggregated VRFs and cognitive function among Chinese older adults is of high relevance for developing the appropriate strategies for the prevention of age-related cognitive impairment and dementia in China.

The ε4 allele of the apolipoprotein E (*APOE*) gene is a known genetic risk factor for AD and global cognitive decline in old age. Evidence from meta-analysis has suggested that the *APOE* ε4 allele was associated with poorer performance on tests of global cognitive function (8, 9). However, most of the previous studies have been carried out among western societies, and very few studies are performed in Chinese older adults. Besides, translocase of outer mitochondrial membrane 40 (*TOMM40*), roughly 15 kb upstream to the *APOE* gene, is responsible for encoding an essential protein for cell viability on the outer mitochondrial membrane. Several single-nucleotide polymorphisms (SNPs) of *TOMM40*, such as rs2075650, have been identified to be associated with the risk of developing AD in different populations, including Han Chinese (10, 11). Linkage disequilibrium (LD) between *TOMM40* rs2075650 and *APOE* rs429358 has been reported in various populations (12–14); thus, the association of *TOMM40* rs2075650 with AD is often considered as a proxy for that of the *APOE* ε4 allele. However, genetic association analyses among *APOE* ε4 non-carriers found that *TOMM40* rs2075650 is still associated with AD (15, 16), suggesting that *TOMM40* might play a role in AD independent of the *APOE* ε4 allele. A few studies have explored the contribution of *TOMM40* rs2075650 to cognitive dysfunction, and the results are inconsistent across different populations. For example, a genome-wide association study has revealed that *TOMM40* rs2075650 was associated with cognitive aging in Swedish cohorts, but not in UK cohorts (17). Furthermore, whether *TOMM40* rs2075650 is associated with global cognitive function independently of the *APOE* ε4 allele is still unclear, especially in Chinese older adults.

Emerging evidence has indicated that VRFs and susceptibility genetic polymorphisms could interact synergistically to exacerbate the cognitive decline. For instance, some studies have reported that VRFs (e.g., diabetes) could interact with *APOE* ε4 to adversely affect global cognitive function (18–20) and specific cognitive domains, such as memory and executive function (21, 22). So far, no study has examined the interaction of VRFs with *TOMM40* rs2075650. Of note, population-based studies that explore potential interactive effects of individual and aggregated VRFs with *APOE* ε4, or with *TOMM40* polymorphisms on cognition are generally lacking in the Chinese population.

Therefore, in this community-based study, we aim to examine (i) the relationship between individual and aggregated VRFs and global cognitive performance; (ii) the relationship of *APOE* genotype and *TOMM40* polymorphisms with cognition; and (iii) the potential interactions between VRFs and two susceptibility polymorphisms on global cognitive function in Chinese older adults. We hypothesized that VRFs, *APOE* ε4, and *TOMM40* G carriers were independently associated with poor cognitive performance and that carrying genetic polymorphisms may strengthen the associations of VRFs with poor cognitive function.

## Methods

### Study Participants

This is a community-based study performed in Beijing, China. The details of this study design and data collection procedures have been fully described previously (23). Briefly, at baseline (2011), 473 participants (age ≥60 years) were randomly selected from permanent residents living in the XiCheng and ChaoYang Districts in Beijing, which represented the old downtown and the newly developed area, respectively. Of these, 40 participants were excluded due to lack of blood samples and an additional 11 participants were excluded owing to probable AD or other types of dementia defined according to the Diagnostic and Statistical Manual of Mental Disorders, Fourth Edition, criteria (DSM-IV), leaving 422 participants for the final analysis of cross-sectional associations.

This study was approved by the Ethics Committee at the Institute of Psychology, Chinese Academy of Sciences, Beijing, China. Written informed consent was given by each participant at each visit.

### Data Collection

At baseline, research assistants with psychological background collected data on demographics or cardiovascular or lifestyle-related factors (e.g., smoking, alcohol consumption, and leisure activity). Physical activity was measured *via* the question “How often do you regularly participate in physical activity at least 20 min per day (never, 1–3 days/week, 4–6 days/week, or every day)?” Psychiatrists conducted the clinical assessment, including health history and use of medications, a routine physical examination, and neuropsychological tests (e.g., Neuropsychiatric Inventory and the Clinical Dementia Rating). Depressive symptoms were assessed using the 30-item Geriatric Depression Scale (GDS-30) (24). Weight and height were measured in light clothes with no shoes. Body mass index (BMI) was calculated as weight (kg) divided by height (m) squared. Arterial blood pressure was measured on the right arm in a sitting position.

After an overnight fast, peripheral blood samples were taken. Plasma glucose, total cholesterol (TC), triglycerides (TG), low-density lipoprotein cholesterol (LDL-C), and high-density lipoprotein cholesterol (HDL-C) were measured using standard enzymatic methods on routine automated chemistry systems. Genomic DNA was isolated from whole blood samples.

### Definitions of Vascular Factors

Smoking and alcohol intake were dichotomized into current and non-current (never and former). Physical inactivity was defined as participating in any physical activity ≤ 3 days/week during leisure time. Obesity was defined as a BMI ≥28 kg/m^2^, a commonly recommended cutoff point for Chinese adults (25). High TC was defined as TC >6.2 mmol/l or use of hypolipidemic drugs, high TG as TG ≥2.3 mmol/l or receiving hypolipidemic drugs, low HDL-C as HDL-C <1.0 mmol/l in men or HDL-C <1.3 mmol/l in women or use of hypolipidemic drugs, and high LDL-C as LDL-C ≥4.1 mmol/l or using hypolipidemic drugs (26). Diabetes was defined as fasting plasma glucose ≥7.0 mmol/l or the current use of oral blood glucose-lowering medications or insulin injections (27). Hypertension was defined as having a self-reported history of hypertension, current use of antihypertensive medication, or blood pressure ≥140/90 mm Hg (28).

### Genotyping

*APOE* and *TOMM40* polymorphisms were genotyped using the Sequenom iPLEX Gold assay and the MassARRAY MALDI-TOF mass spectrometry platform (San Diego, CA, USA). The *APOE* genotype was dichotomized as carriers vs. non-carriers of the *APOE* ε4 allele, and *TOMM40* rs2075650 as carriers vs. non-carriers of the G allele.

### Definitions of Covariates

The presence of depressive symptoms was defined as the GDS-30 score >10. Heart disease was defined as having a myocardial infarction, atrial fibrillation, heart failure, or angina.

### Assessment of Global Cognitive Function

Global cognitive functioning was assessed with the Chinese version of the Mini-Mental State Examination (MMSE). Most items of the MMSE test were translated literally from the original version without modification, while some items were adopted to meet the Chinese cultural context (29). It is a validated and widely used test of assessing global cognitive function with orientation, memory, attention-calculation, language, and registration components. The total MMSE score ranges from 0 to 30, with higher scores denoting better cognitive performance.

### Statistical Analysis

The participants' characteristics by *APOE* ε4 and *TOMM40* G carrier status were compared using independent *t*-tests for continuous variables and χ^2^ tests for categorical variables.

We dichotomized each of the VRFs, *APOE* ε4 and *TOMM40* G carrier status, depressive symptoms, and heart disease as presence vs. absence. Multivariable linear regression models were used to analyze the associations of the MMSE score with various factors. Firstly, we estimated the β coefficients and 95% confidence interval (*CI*) of the MMSE score associated with individual and aggregated VRFs, *APOE*, and *TOMM40* polymorphisms. The aggregated VRF measure was calculated by summing up the total number of concurrently presented putative VRFs that were associated with the MMSE score in multivariable linear regression analysis (30). In order not to miss any potential risk factors associated with the MMSE score, we used a conservative *p*-value of 0.35 for the inclusion of the putative VRFs, as previously reported (31, 32). The aggregated VRF was treated as either a continuous or categorical variable in the analyses.

We also assessed the interactions of individual and aggregated VRFs with genetic polymorphisms by simultaneously including the two independent variables and their cross-product terms in the same model. To examine the joint effects of VRFs with a genetic polymorphism, we divided subjects into 4 groups: those with neither VRFs nor the genetic polymorphism (reference), with only VRFs (e.g., diabetes), with only the genetic polymorphism (e.g., *APOE* ε4 allele), or with both VRFs and genetic polymorphism.

We reported results from two models: model 1 was adjusted for demographic variables (i.e., age, sex, and education) and model 2 was further adjusted for the presence of depressive symptoms and heart disease. Given the moderate LD between *APOE* rs429358 and *TOMM40* rs2075650 in Han Chinese people (12), in models 1 and 2, we additionally adjusted for each other to verify their independent associations with the MMSE score. IBM SPSS Statistics 21.0 for Windows (IBM Corp., Armonk, NY) was used for all statistical analyses.

## Results

### Characteristics of the Study Participants

The characteristics of the study participants were summarized as shown in Table 1. Of the 422 participants, the mean age was 69.2 (SD, 6.2) years, 54.3% were women, and 95.5% were Han Chinese. Among these participants, 68 (16.1%) carried at least one *APOE* ε4 allele and 81 (19.2%) carried *TOMM40* G. The frequency distributions of the two genes were similar to the previous report from the large-scale study in Beijing (33). The *APOE* ε4 carriers and non-carriers did not differ significantly in the mean age and years of education, or in the distribution of sex, current smoking, alcohol intake, physical inactivity, obesity, high cholesterol, high triglycerides, low HDL-C, high LDL-C, hypertension, diabetes, depression, and heart disease (for all comparisons, *p* > 0.05). Furthermore, there were no significant differences between *TOMM40* G carriers and non-carriers on foregoing factors (for all comparisons, *p* > 0.05), except for high LDL-C (*p* = 0.05). The mean MMSE score was 27.1 (SD, 3.5), and there were no significant differences in MMSE score between *APOE* ε4 carriers and non-carriers (*p* = 0.66) and between *TOMM40* G carriers and non-carriers (*p* = 0.11).

**Table 1:** Characteristics of the study participants at baseline (*n* = 422).

**Baseline**	**Total**	***APOE*** **ε4 carrier**			***TOMM40*** **G carrier**		
**Characteristics**	**Sample**	**No**	**Yes**	***p*-value**	**No**	**Yes**	***p*-value**
No. of subjects	422	354	68		341	81	
Age, y, mean (SD)	69.0 (6.2)	69.8 (6.2)	70.14 (6.5)	0.68	69.8 (6.3)	70.1 (6.3)	0.76
Women, n (%)	229 (54.3)	194 (55.0)	35 (50.7)	0.52	184 (54.0)	45 (55.6)	0.80
Education, y, mean (SD)	12.5 (4.8)	12.5 (4.8)	12.7 (4.5)	0.78	12.6 (4.7)	12.3 (4.9)	0.64
Current smoking, n (%)	52 (12.3)	45 (12.7)	7 (10.1)	0.55	46 (13.5)	6 (7.4)	0.13
Current drinking, n (%)	50 (11.8)	42 (11.9)	8 (11.6)	0.94	39 (11.4)	11 (13.6)	0.59
Physical inactivity, n (%)	97 (23.0)	78 (22.1)	19 (27.5)	0.33	77 (22.6)	20 (24.7)	0.69
Obesity, n (%)	66 (15.6)	56 (15.9)	10 (14.5)	0.77	51 (15.0)	15 (18.5)	0.43
High cholesterol, n (%)	95 (22.5)	76 (21.5)	19 (27.5)	0.30	71 (20.8)	24 (29.6)	0.09
High triglycerides, n (%)	110 (26.1)	88 (24.9)	22 (31.9)	0.23	83 (24.3)	27 (33.3)	0.10
Low HDL-C, n (%)	160 (37.9)	134 (38.0)	26 (37.7)	0.97	104 (37.8)	43 (35.8)	0.76
High LDL-C, n (%)	96 (22.7)	76 (21.5)	20 (29.0)	0.18	71 (20.8)	25 (30.9)	0.05
Hypertension, n (%)	254 (60.2)	214 (60.6)	40 (58.0)	0.68	204 (59.8)	50 (61.7)	0.75
Diabetes, n (%)	82 (19.4)	65 (18.4)	17 (24.6)	0.23	64 (18.8)	18 (22.2)	0.48
Depressive symptoms, n (%)	59 (14.0)	46 (13.0)	13 (18.8)	0.20	45 (13.2)	14 (17.3)	0.34
Heart disease, n (%)	69 (16.4)	58 (16.4)	11 (15.9)	0.92	56 (16.4)	13 (16.0)	0.94
MMSE score, mean (SD)	27.1 (3.5)	27.2 (3.4)	26.9 (3.8)	0.66	27.3 (3.3)	26.6 (4.1)	0.11

*APOE, apolipoprotein E; TOMM40, translocase of outer mitochondrial membrane 40; HDL-C, high-density lipoprotein cholesterol; LDL-C, low-density lipoprotein cholesterol*.

### Associations of Individual and Aggregated VRFs With MMSE Score

Associations of individual VRFs with MMSE score were summarized as shown in Table 2. Specifically, physical inactivity was significantly associated with a low MMSE score in model 1 (*p* = 0.01), and the results remained significant when depression and heart disease were further adjusted for in model 2 (*p* = 0.02). Similarly, diabetes was also significantly associated with a low MMSE score in model 1 (*p* = 0.001), and the significance did not change at all in model 2. No other VRFs were significantly associated with the MMSE score.

**Table 2:** Associations of MMSE score with vascular risk factors (*n* = 422).

**Vascular risk factors**	*****β***** **coefficient (95% confidence interval), MMSE score**			
	**Model 1^a^**	***p***	**Model 2^a^**	***p***
Current smoking	−0.50 (−1.32, 0.33)	0.24	−0.52 (−1.33, 0.31)	0.22
Alcohol intake	0.19 (−0.64, 1.01)	0.66	0.21 (−0.61, 1.02)	0.62
Obesity	−0.12 (−0.82, 0.58)	0.74	−0.14 (−0.84, 0.55)	0.69
Physical inactivity	−0.77 (−1.36, −0.17)	0.01	−0.70 (−1.30, −0.11)	0.02
High total cholesterol	−0.15 (−0.76, 0.47)	0.65	−0.21 (−0.83, 0.41)	0.51
High triglycerides	−0.11 (−0.69, 0.47)	0.71	−0.16 (−0.74, 0.42)	0.59
Low HDL-C	−0.05 (−0.58,0.48)	0.86	−0.11 (−0.64, 0.42)	0.69
High LDL-C	−0.24 (−0.86, 0.37)	0.43	−0.31 (−0.92, 0.31)	0.33
Hypertension	0.41 (−0.10, 0.93)	0.12	0.36 (−0.16, 0.88)	0.18
Diabetes	−1.18 (−1.81, −0.55)	0.001	−1.14 (−1.78, −0.51)	0.001

*HDL-C, high-density lipoprotein cholesterol; LDL-C, low-density lipoprotein cholesterol*.

a Model 1 was controlled for age, sex, and education, and

b *model 2 was additionally controlled for the presence of depressive symptoms and heart disease*.

When a conservative *p-*value of 0.35 was used to include all the potential risk factors, four putative VRFs (i.e., current smoking, physical inactivity, high LDL-C, and diabetes) were counted to calculate the aggregated VRF score (Table 2). As a continuous variable (0–4), an increasing VRF score was significantly associated with a lower MMSE score (*p* = 0.001) (Table 3). As a categorical variable (categorized into 0, 1, and ≥2), having 2 or more of these VRFs, in comparison with having none, was significantly associated with a lower MMSE score, even when further adjusting for the presence of depressive symptoms and heart disease (*p* = 0.001) (Table 3, models 1 and 2).

**Table 3:** Associations of MMSE score with aggregated vascular risk factors (*n* = 422).

**No. of vascular risk factors^a^**	*****β***** **coefficient (95% confidence interval), MMSE score**			
	**Model 1^b^**	***p***	**Model 2^b^**	***p***
As a continuous variable (0–4)	−0.59 (−0.89, −0.29)	0.001	−0.60 (−0.90, −0.30)	0.001
**As a categorical variable**				
0 (*n* = 185)	0.00 (reference)		0.00 (reference)	
1 (*n* = 162)	−0.11 (−0.65, 0.43)	0.70	−0.12 (−0.67, 0.42)	0.66
≥2 (*n* = 75)	−1.45 (−2.15, −0.76)	0.001	−1.49 (−2.19, −0.79)	0.001
*P* for linear trend	0.001		0.001	

a *Vascular risk factors included current smoking, physical inactivity, high LDL-C, and diabetes*.

b *Model 1 was controlled for age, sex, education, and model 2 was additionally controlled for the presence of depressive symptoms and heart disease*.

### Associations of Genetic Polymorphisms With MMSE Score

Carrying the *APOE* ε4 allele was not significantly associated with MMSE score after adjusting for age, sex, education, and *TOMM40* in model 1 (β: 0.41, 95% *CI*: −0.62 to 1.44, *p* = 0.44), and the results were similar when depressive symptoms and heart disease were additionally controlled for in model 2 (β: 0.47, 95% *CI*: −0.55 to 1.50, *p* = 0.36). Carrying the *TOMM40* G allele was marginally associated with a low MMSE score when age, sex, education, and *APOE* were adjusted for (β: −0.84, 95% *CI*: −1.79 to 0.10, *p* = 0.08), and the results were similar in the fully adjusted model (β: −0.87, 95% *CI*: −1.81 to 0.08, *p* = 0.07).

### Interactions Between VRFs and Genetic Polymorphisms

First, we analyzed the interactions of aggregated VRFs with *APOE* genotype and *TOMM40* polymorphisms. We did not detect a significant interaction between aggregated VRFs and *APOE* ε4 in model 1 and the fully adjusted model (all *p* for interaction > 0.05). However, a marginally significant interaction between aggregated VRFs and the *TOMM40* polymorphism was detected (*p* = 0.06), which became statistically evident when further adjusting for depressive symptoms and heart disease (*p* = 0.05). Further analysis stratified by *TOMM40* indicated that among *TOMM40* G carriers, having 1 or more of these VRFs was potentially correlated with a lower MMSE score in model 1 (β: −1.38, 95% *CI*: −2.80 to 0.03, *p* = 0.05). However, among *TOMM40* G non-carriers, an association of aggregated VRFs with a lower MMSE score was not significant (β: −0.28, 95% *CI*: −0.82 to 0.28, *p* = 0.31). The results were similar in the fully adjusted model. These associations detected in foregoing genetic analyses were independent of the *APOE* ε4 carrier status.

**Figure 1:**
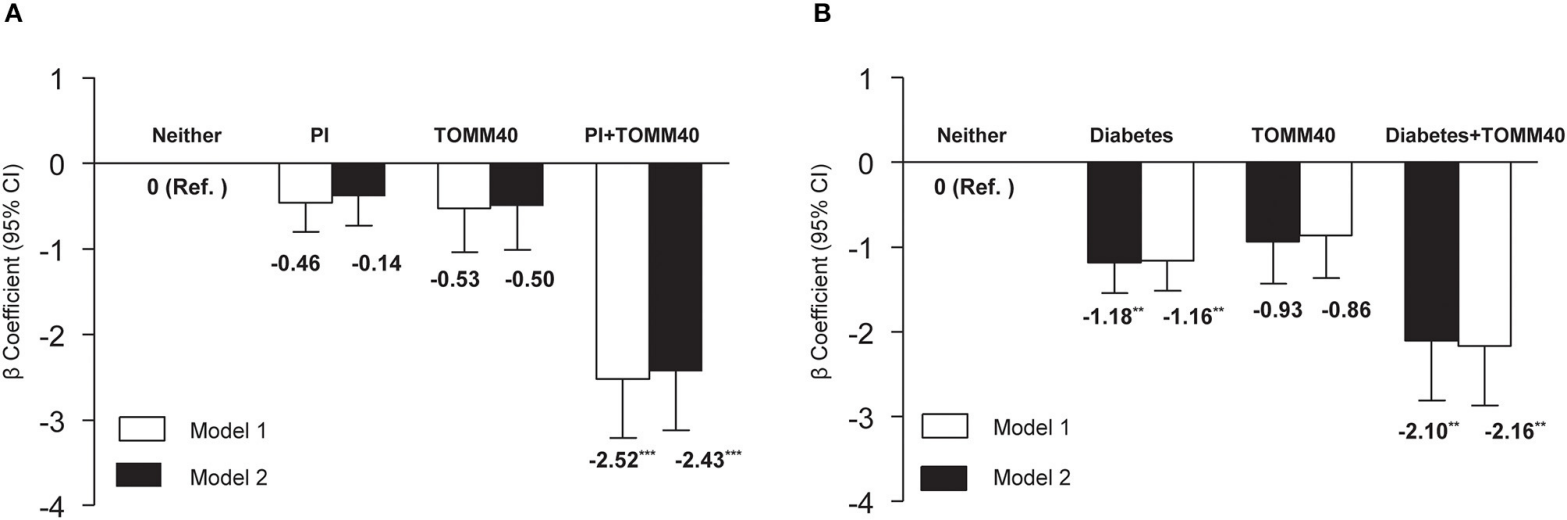
**(A)** Joint effects of physical inactivity (PI) and *TOMM40* polymorphisms, and **(B)** diabetes and *TOMM40* polymorphisms on MMSE score. Model 1 was controlled for age, sex, education, and *APOE* polymorphism, and model 2 was additionally controlled for the presence of depressive symptoms and heart disease. **p < 0.01; ***p < 0.001.

We also explored the interactions of individual VRFs with *APOE* ε4, or with *TOMM40* G. Interactions between individual VRFs and *APOE* ε4 were not significant after *TOMM40* adjustment in model 1 and fully adjusted model (all *p* > 0.05). However, the interaction between physical inactivity and *TOMM40* G was statistically significant (*p* = 0.01). The joint-effect analysis showed that compared to people with neither physical inactivity nor *TOMM40* G, individuals with only physical inactivity or only *TOMM40* G did not have a significantly lower MMSE score, but people having both physical inactivity and *TOMM40* G had a markedly lower MMSE score in model 1 (β: −2.52, 95% *CI*: −3.88 to −1.16, *p* = 0.001), and the results were similar in the fully adjusted model (Figure 1A). The results also revealed a significant interaction between diabetes with *TOMM40* G (*p* = 0.03). Further analysis indicated that compared to people with neither diabetes nor *TOMM40* G, individuals with diabetes alone had a significantly lower MMSE score in model 1 (β: −1.18, 95% *CI*: −1.189 to −0.47, *p* = 0.001) and people having both diabetes and *TOMM40* G had a markedly lower MMSE score in model 1 (β: −2.10, 95% *CI*: −3.49 to −0.71, *p* = 0.003), and the results were similar in the fully adjusted model (Figure 1B); moreover, these associations were independent of the *APOE* polymorphism. No other individual VRFs were found to interact with *TOMM40* G to affect cognitive performance.

## Discussion

The primary aim of the current study was to investigate the associations of VRFs, *APOE*, and *TOMM40* polymorphisms with cognitive performance in Chinese older adults who were free of dementia, with a focus on potential interactions between VRFs and the two genetic polymorphisms. We found that physical inactivity and diabetes were independently associated with poor global cognitive performance. When four putative VRFs (i.e., current smoking, physical inactivity, high LDL-C, and diabetes) were aggregated, an increasing number of concurrently having these factors was associated with a decreasing MMSE score in a dose–response manner. Furthermore, we detected an interaction between the aggregated VRFs and *TOMM40* G, such that having 1 or more VRFs, in combination with *TOMM40* G, was associated with markedly low cognitive function in Chinese older adults. We also revealed interactions of physical inactivity with *TOMM40* G, and diabetes with *TOMM40* G independent of the *APOE* polymorphism, such that *TOMM40* G in combination with either physical inactivity or diabetes was associated with a lower MMSE score.

### Associations of VRFs With Global Cognitive Function

Physical activity has been shown to benefit cognition in older adults (34). For example, a population-based study with 104,909 participants from 20 countries showed that physical activity was associated with global cognitive performance (35). In line with previous literature, we found that low physical activity was associated with poor cognitive performance in Chinese older adults (36). In addition, diabetes was reported to be associated with deficits in global cognitive function as assessed by the MMSE in a previous study (37). We replicated this result by detecting an association of diabetes with a lower MMSE score in older adults. However, given the relatively small sample of our study and the potential false-positive associations due to multiple comparisons, caution is needed when interpreting these results.

Some individual VRFs (e.g., smoking and hypertension) have been linked with poor cognitive performance in other studies (3, 38, 39); however, these factors were not associated with MMSE score in the present study. It has been reported that the associations of some VRFs, such as hypertension, obesity, and high cholesterol with cognitive function, largely depend on age. That is, having these factors in midlife or young–old age, but not necessarily in very old, was associated with age-related cognitive impairment and dementia (40–42). Indeed, 51.8% of the participants in this study were aged over 70 years. This might partly contribute to the lack of positive associations between some VRFs and poor cognitive function in our study. Further large prospective cohort studies that consist of different age groups are necessary to further clarify the associations of VRFs with cognitive decline.

VRFs are often coexisting together in middle-aged or older populations. Accumulating evidence showed that people who are exposed to multiple VRFs simultaneously often perform much worse on different cognitive tests (7, 43) and have a much higher risk of developing dementia than those who have a single VRF (44). However, most of these studies are performed in western countries. Our results extend prior work by showing that aggregated VRFs are associated with poor global cognitive function among community-dwelling Chinese older adults. These results imply that early interventions targeting multiple modifiable risk factors among cognitively healthy older adults may partly ameliorate cognitive decline and may delay the onset of dementia syndrome.

### Associations of Genetic Polymorphisms With Cognitive Function

Carrying the *APOE* ε4 allele in our study participants was not associated with the MMSE score. Although some studies from western countries (e.g., Northern Europe) have found *APOE* ε4-related decline in global cognitive function (45, 46), a population-based study carried out in Chinese older adults, actually, failed to observe a significant association of *APOE* ε4 with MMSE score (47). We speculate that the ethnic differences in genetic susceptibility may partially contribute to the discrepancy of research findings across studies. Indeed, compared to the proportion of people carrying *APOE* ε4 in the Nordic population (around 25–30%), the proportion in our study is relatively low (16.1%), and only 1.4% were homozygous for ε4. In addition, episodic memory and executive functioning were reported to be particularly affected by carrying the ε4 allele (48–50). Thus, global cognitive function as assessed with the MMSE may not be as sensitive to the influence of the *APOE* ε4 allele. Further large-scale population-based studies are needed to investigate the associations between other cognitive domains and the *APOE* ε4 allele in dementia-free Chinese older adults.

Our results extend prior work by showing that *TOMM40* G carriers showed poor global cognitive function independent of the *APOE* ε4 allele in Chinese older adults. However, large-scale population-based studies are recommended to further examine the relationship between *TOMM40* G and cognitive function. *TOMM40* is mainly responsible for mitochondrial function. Mitochondrial dysfunction is implicated in neurodegenerative disorders, and *TOMM40* may be influential in this regard by influencing mitochondrial neurotoxicity. Mitochondrial dysfunction is common in AD, and it may be the primary event that causes deposition of Aβ deposition, degeneration of synapses, and neuronal death in AD (51, 52). Future AD therapies aimed at boosting mitochondrial function may offer an alternative treatment strategy.

### Interactions Between VRFs and Genetic Polymorphisms

We detected interactions of VRFs (i.e., physical inactivity, diabetes, and aggregated VRFs) with *TOMM40* G on global cognitive performance. The interactive effects suggest that carrying *TOMM40* G, in combination with VRFs (i.e., physically inactive, diabetes), and particularly aggregated multiple VRFs, has substantially poorer performance in a global cognitive test. This is the first study to reveal synergistic effects of individual and aggregated VRFs with *TOMM40* G on global cognitive function. This finding deserves further confirmation in different ethnic groups in China. The biological mechanisms behind the interactions are not fully understood. Previous research found that the *TOMM40* rs2075650 G allele, independent of the *APOE* ε4 allele, was associated with depression and obesity, which are risk factors for dementia (53, 54). *TOMM40* rs2075650 was also found to be specifically associated with bilateral hippocampus and right amygdala volume (55). Furthermore, evidence has indicated that VRFs can accelerate cognitive decline by aggravating mitochondrial damage (56). It is possible that *TOMM40* in combination with different VRFs influences mitochondrial function and further leads to brain structure changes and eventually affects human behavior (52). The mechanisms for these VRFs-*TOMM40* interactions deserve further exploration. Our results highlight the potential importance of interventions by targeting multiple VRFs to maintain cognitive function, especially among *TOMM40* G carriers.

### Strengths and Limitations

This is one of the first community-based studies of Chinese older adults to examine the associations of VRFs and genetic polymorphisms (*APOE* and *TOMM40*) with global cognitive performance as assessed with the MMSE test. We randomly recruited the study sample from the communities. However, the limitations of our study deserve mention. Firstly, we cannot determine the temporal and causal relationships from a cross-sectional study, and the finding of any association might be subject to bias due to selective survival. However, selective survival is likely to dilute the true associations given that exposures (VRFs and *APOE* ε4) and outcomes (poor cognitive performance) are known to be associated with mortality. Secondly, the study sample is relatively small, especially for genetic studies, which might have inadequate power to detect a weakly-to-moderately strong association between those factors and the MMSE score. Thirdly, MMSE is a validated and widely used test of global cognitive functioning with orientation, memory, attention calculation, language, and registration components; however, it lacks specific items that test executive functions and psychomotor speed. The cognitive domains that are often impaired by exposures to VRFs and related cerebrovascular disease therefore might not be sensitive enough to detect cognitive impairment. Thus, it is worth examining the relationship of VRFs and genetic polymorphisms with different cognitive domains in future studies. Finally, physical activity was assessed based on self-reported information, and we did not have detailed information regarding frequency, duration, intensity, types, or energy expenditure of physical activity. Thus, it could be difficult to define physical activity accurately. Future studies that use objective measurements of physical activity, such as accelerometers and measures of aerobic fitness, as well as self-reported questionnaires that provide proxies of energy expenditure may help more properly address this issue.

## Conclusion

This community-based study provides evidence supporting associations between individual and aggregated VRFs with poor cognitive function. In addition, we found a marginally significant association of *TOMM40* with cognitive performance independent of the *APOE* gene and further detected interactions between VRFs (e.g., physical inactivity, diabetes, and aggregated VRFs) and *TOMM40* G but not for the *APOE* ε4 variant. Research efforts to identify therapeutic approaches for preclinical and clinical dementia have so far largely focused on the amyloid pathway, but almost all the clinical trials have yielded limited benefits. These results imply that early interventions by targeting multiple modifiable risk factors among cognitively healthy older adults may partly ameliorate cognitive decline, therefore delaying the onset of the dementia syndrome, especially for carriers of risk genes. Large-scale community-based longitudinal studies and interventions are needed to further clarify the causal relationships and mechanisms between VRFs and cognitive function and their interactive effects with genetic polymorphisms on cognition.

## Data Availability Statement

The datasets generated and analyzed during the current study are not available publicly as ethical clearance was not obtained to share data publicly. However, the data are available from the corresponding author on reasonable request.

## Ethics Statement

This study was approved by the Ethics Committee at the Institute of Psychology, Chinese Academy of Sciences, Beijing, China. Written informed consent was given by each participant at each visit.

## Author Contributions

WG and JL developed the research questions and designed the study. WG analyzed the data and wrote the manuscript. JL was the principal investigator of this project. JL and CQ supervised the study. JL, CQ, and QS critically revised the paper. All authors approved the final version of the paper.

## Conflict of Interest

The authors declare that the research was conducted in the absence of any commercial or financial relationships that could be construed as a potential conflict of interest.
